# *Neisseria meningitidis* as a Cause of Septic Arthritis: An Unusual Case of Periprosthetic Joint Infection

**DOI:** 10.1155/2020/8431019

**Published:** 2020-03-10

**Authors:** A. Mc Carthy, J. M. Broderick, A. P. Molloy

**Affiliations:** Department of Trauma and Orthopaedic Surgery, St. Vincent's University Hospital, Dublin, Ireland

## Abstract

One of the most feared complications after arthroplasty is infection due to its significant impact on patient morbidity. Infection may transfer to the joint at the time of surgery or be seeded, haematologically, to the prosthetic joint from another infection source. In this case, a 72-year-old female presented with symptoms of septic arthritis seven years after her original arthroplasty surgery. At presentation, she denied trauma and any comorbidity which would predispose her to infection. Culturing of samples taken revealed the patient was infected with *Neisseria meningitidis*, and the patient underwent a DAIR procedure. She continued postoperative long-term antimicrobial therapy with resolution of her infection. Follow-up at one year showed complete resolution of the patient's illness with a return to premorbid baseline. To our knowledge, this is the third reported case of septic arthritis caused by *Neisseria meningitidis* in a prosthetic joint in the literature.

## 1. Introduction

Prosthetic joint infection (PJI) is a potentially devastating complication of joint arthroplasty [1]. It is reported to occur in approximately 1% of total hip arthroplasty (THA) and 1–4% of total knee arthroplasty (TKA) and is the second most common cause for revision after mechanical loosening [2, 3]. PJI may be acute (4–6 weeks), delayed (6–24 months), or late (>2 years) [4]. Late PJIs often have an acute onset and are due to haematogenous spread in a previously asymptomatic joint [4].

The most common causative organism is *Staphylococcus aureus* [5], which has been shown to have a 30–40 percent risk of haematogenous seeding of in situ arthroplasties [4]. However, coagulase-negative staphylococci, *Streptococcus* species, *Enterococcus* species, and aerobic Gram-negative bacilli are also commonly isolated [4].

Rarer PJIs include organisms such as *Neisseria meningitidis*. *Neisseria meningitidis* is a fastidious, encapsulated, aerobic, Gram-negative diplococcus which remains the leading cause worldwide of bacterial meningitis, despite the availability of effective antibiotics and a broad understanding of the pathogenesis [6]. According to current microbiological and epidemiological research, the human species remains the only natural host for meningococcus [6]. *Neisseria meningitidis* has a wide spectrum of clinical manifestations from life-threatening sepsis to asymptomatic colonisation of the nasopharynx in endemic periods of infection [6]. Rarely, it can lead to localised forms of infection such as pneumonia, pericarditis, sinusitis, and septic arthritis. Based on our search of the scientific databases including PubMed, EMBASE, and Google Scholar, only two previous case reports of a PJI due to *Neisseria meningitidis* exist [7, 8]. We thus present a new case of this rare infection.

## 2. Case Report

A 72-year-old Caucasian female patient presented to the emergency department with a two-day history of worsening left knee pain, swelling and erythema. She has associated malaise, diaphoresis, and pyrexia. She denied headache, neck stiffness, skin rash, recent sick contacts, and foreign travel. Her medical history was significant for a left TKA performed 7 years previously with a cemented, cruciate-retaining prothesis. There had been no issues with regard to wound ooze or infection at the time of surgery, and her TKA had been functioning well up until this presentation. The patient denied trauma to the knee, a history of diabetes, or previous joint infections. Socially, the patient was single and lived alone; she reported having been in contact with her neighbour's child seven days prior when she had babysat but neither the child nor anyone in the neighbour's house was unwell.

On physical exam, she had a left knee effusion with erythema and limited, painful range of motion. Neurological exam was normal. There was no evidence of skin rash, neck stiffness, cardiac murmurs, respiratory disease, or visual changes. Vitals at presentation included a temperature of 38.6 degree Celsius, heart rate of 78 bpm, blood pressure of 120/64 mmhg, and her saturations were 99% on room air. CRP was 348 mg/L and WCC was 9.2 × 10∼9/l, with neutrophils of 7.3 × 10.9/l. On aspiration of her knee joint under aseptic conditions, approximately 15 ml of purulent fluid was obtained and sent for Gram stain, culture, and sensitivity as well a white count and differential. X-Ray of the left knee showed a well-aligned prosthesis with no evidence of loosening or osteolysis.

On the following day, the patient underwent a DAIR (debridement, antibiotics, and implant retention) procedure with isolated polyethylene tibial insert exchange. Tissue samples were sent for culture and sensitivity. Microbiology and infectious diseases consults were also sought as per hospital protocol. Day 1 after the procedure, the microbiology department contacted the orthopaedic team to advise that the Gram stain had shown Gram-negative diplococci, and the aerobic bottle of the blood cultures and the samples from the theatre had grown *Neisseria Meningitidis*. Culture was grown using an NYC agar medium. The patients' IV flucloxacillin was continued, and IV cefotaxime was added to the regime as per the sensitivities tested in the lab and on consultation with infectious diseases and microbiology. All members of the surgical and anaesthetic teams who had been present for the DAIR were required to take a prophylactic dose of ciprofloxacin. Both the orthopaedic and microbiology teams discussed the diagnosis with the patient, and the microbiology team was involved in contact tracing to ensure adequate prophylaxis for close contacts of the patient.

The patient remained in the hospital for 11 days, during which time she continued to receive antibiotics and input from the multidisciplinary team, particularly physiotherapy. On discharge, she was mobilising with a rollator zimmer frame; her baseline was independent mobilisation.

At discharge, her CRP was 32 mg/L, WCC were 5.2 × 10∼9/l, and neutrophils were 1.0 × 10∼9/l, and the patient was referred to Outpatient Antibiotic Therapy services (OPAT). The microbiology and infectious diseases services advised ceftriaxone 2 g OD for six weeks postdischarge followed by six weeks of oral amoxicillin. The decision for this antibiotics regime was made by the head of infectious diseases in St. Vincent's University Hospital, who consulted on and was heavily involved in the case. After discharge, the patient went to convalescence and was reviewed weekly by the infectious diseases team and every two weeks by the orthopaedic team.

At the latest follow-up which was one year after initial presentation, the patient was mobilising independently, and her knee was pain-free and stable. Her range of motion was documented at 0–100 degrees. WCC were 4.2 × 10∼9/l, and neutrophils were 2.8 × 10∼9/l with a CRP was 2.0 mg/L. She required a full meningococcal vaccination at the end of her antibiotic regime. It was at this follow-up that the patient gave permission to the team to publish her case report.

## 3. Discussion

The projected burden of osteoarthritis and the need for definitive surgical management with arthroplasty continues to increase [9]. Although TKA provides many benefits including restoration of function and reduction in pain, like all surgeries, it is not without the risk of complications. Complications after arthroplasty include ongoing long-term pain, periprosthetic fracture, and PJI [11].

PJI has a reported incidence of 1–4% in TKA and is the second most common cause for revision surgery after aseptic loosening. Revision in the setting of PJI is typically performed as a two-stage procedure [11]. Stage 1 involves implant removal, soft tissue debridement, and insertion of a temporary, antibiotic-loaded spacer. The patient then undertakes a prolonged course of antibiotics which is followed by an antibiotic-free period. Once there is evidence of infection eradication, a new prothesis is reinserted as a second procedure. In some units, this revision surgery is performed in a single one-stage revision. However, the reported success rates of upon revision surgery in the setting of PJI vary, and functional outcomes are often worse than for primary arthroplasty [11]. Both the infection and its treatment represent a significant physical and psychological burden for both the patient and family. There is also a substantial financial cost to the healthcare system. For these reasons, the DAIR procedure may be considered in patients without signs of septic loosening who has had the infection less than three weeks, and the pathogen isolated can be eliminated by antimicrobial agents [12].

In our case, a rare pathogen, *Neisseria meningitidis*, was isolated from the patient. There are an estimated 1.2 million cases of meningococcal infection per year with a death toll of 135000 worldwide [12]. While peak incidence of infection occurs among infants and adolescents, over one-third of sporadic cases seen worldwide occur in adults [6, 12]. The ability of *Neisseria meningitidis* to infect the prosthetic joint will be dependent upon the affinity of the osteoarticular tissue, the type of prothesis employed, the amount of residual joint tissue, and the formation of biofilm. Both encapsulated and nonencapsulated forms of *Neisseria meningitidis* can produce biofilms, and these are the key to bacteria's ability to colonise nonbiological surfaces [6, 12].

Clinically, there are three syndromes associated with *Neisseria meningitidis*-acute meningococcaemia, transient meningococcaemia, and chronic meningococcaemia [6–8, 12]. Acute meningococcaemia is associated with sepsis or meningitis, while chronic meningococcaemia presents with intermittent episodes of fever, rash, arthralgia, and maculopapular rash. Transient meningococcaemia is characterised by fever and rash which evolve over a 2–5 day period with *Neisseria meningitidis* considered a rare finding on the blood culture isolate [6, 12].

Three clinical scenarios regarding meningococcal arthritis were described by Schaad et al. [13]; the most common being arthritis as a complication of acute meningococcal disease. It has an immunological basis, affects the knees in 95% of cases, and is usually polyarticular [13]. The second clinical type involved patients with chronic meningococcaemia with accompanying arthralgia; a hypersensitivity state was theorised but not proven [13]. The third category includes patients with primary meningococcal arthritis and septic arthritis without the classical syndrome of meningococcaemia [13]. This affects the large joints almost exclusively and is monoarticular in two-thirds of the cases.

Upon extensive review of the literature, only two other previous cases of septic arthritis in a periprosthetic joint have been documented in the literature [7, 8], making this the third reported case of primary meningococcal infection in a prosthetic joint. The rarity of this presentation is most likely due to the fact that *Neisseria meningitidis* is more associated with systemic infections and less commonly causes localised infections such as septic arthritis or pericarditis [6].

The previous cases were published by Vikram in 2001 [8] and Carral et al. in 2017 [7]. Vikram's case described a Y serogroup form of *Neisseria Meningitidis* which infected a three-year-old prosthesis in an 80-year-old female; management included prosthesis retention and a six-week course of IV antibiotics [8]. The case demonstrated in the literature by Carral et al. [7] was that of a 78-year-old female who presented seven months after left knee arthroplasty. Her cultures were positive for *Neisseria meningitidis* serogroup B. Treatment included arthroscopic debridement with implant retention and a 12-week antibiotic regime; 3 weeks of IV ceftriaxone followed by the oral administration of ciprofloxacin 750 mg daily [7].

In the previous two cases, both author groups supposed that the most likely route for pathogen entry as by haematogenous spread [7, 8]. We believe that a similar route was provided for the pathogen in our case. However, unlike both previous cases where the patient had been involved in a prodromal trauma, our patient denied all history and knowledge of trauma to the knee. Regarding treatment of these cases it must be acknowledged that the traditional means of treating prosthetic joint infections involves a two-stage revision technique [12]. However, in the two cases described in the literature as well as our own, the prosthesis was retained. In our case and similar to that described by Vikram [8], the prosthesis was retained as there was no evidence of loosening, surgical intervention was completed within 24 hours of presentation, and the patient had a rapid response to surgical drainage and iv antibiotics [8, 12].

## 4. Summary

Prosthetic joint infection is a rare but devastating complication of arthroplasty and can occur in both the acute and late phases postoperatively. This case report details a case in which a 72-year-old lady presented 7 years after her original TKR with symptoms of septic arthritis and was treated with a DAIR procedure and long-term antibiotics which covered her pathogen *Neisseria meningitidis*. This case identifies a rare cause of PJI without predisposing risk factors caused by an organism normally considered to have a low affinity for infection of joint tissue and abiotic surfaces.
